# Influence of Chromosome 9p21.3 rs1333049 Variant on Telomere Length and Their Interactive Impact on the Prognosis of Coronary Artery Disease

**DOI:** 10.3390/jcdd10090387

**Published:** 2023-09-07

**Authors:** Andrea Borghini, Antonella Mercuri, Jonica Campolo, Marina Parolini, Rudina Ndreu, Stefano Turchi, Maria Grazia Andreassi

**Affiliations:** 1 CNR Institute of Clinical Physiology, 56124 Pisa, Italy; nella@ifc.cnr.it (A.M.); rudina.ndreu@cnr.it (R.N.); stefano.turchi@cnr.it (S.T.); andreassi@ifc.cnr.it (M.G.A.); 2 CNR Institute of Clinical Physiology, ASST Grande Ospedale Metropolitano Niguarda, 20162 Milan, Italy; jonica.campolo@cnr.it (J.C.); marina.parolini@cnr.it (M.P.)

**Keywords:** chromosome 9p21.3 rs1333049 variant, leucocyte telomere length, coronary artery disease, major adverse cardiovascular events

## Abstract

Background: Both telomere shortening and the chromosome 9p21.3 (Chr9p21) rs1333049 (G/C) variant are involved in coronary artery disease (CAD) risk, likely affecting mechanisms related to cell cycle arrest and vascular senescence. The aim of the study was to examine the link between Chr9p21 rs1333049 variant and leucocyte telomere length (LTL), as well as their interactive effect on the risk of major adverse cardiovascular events (MACEs). Methods: A cohort of 472 patients with angiographically proven and clinically stable CAD were included in the study. At baseline, the LTL, biochemical parameters, and genotype analysis of Chr9p21 rs1333049 variant were measured in all patients. The primary endpoint of this study was the occurrence of MACE defined as a composite of coronary-related death, nonfatal MI, and coronary revascularization. Results: On multivariable linear regression analysis, age (*p* = 0.02) and Chr9p21 rs1333049 variant (*p* = 0.002) were the only independent predictors of LTL levels. Carriers of the CC genotype of this SNP had shorter telomeres than GC carriers (*p* = 0.02) and GG carriers (*p* = 0.0005). After a follow-up with a mean period of 62 ± 19 months, 90 patients (19.1%) had MACE. Short LTL was an independent prognostic factor of MACE incidence (HR:2.2; 95% CI: 1.3–3.7; *p* = 0.005) after adjustment for potential confounders. There was a significant interaction (*p* = 0.01) between the LTL and rs1333049 variant, with patients with risk-allele C and short LTL having a higher risk (HR:5.8; 95% CI: 1.8–19.2; *p* = 0.004). Conclusion: A strong relationship between LTL and Chr9p21 rs1333049 variant was identified, and they interactively affect the risk of poor prognosis in CAD patients.

## 1. Introduction

Telomeres are specific repeat sequences (TTAGGG)n located at chromosome ends, which inexorably shorten with every cell division because of end replication mispairing and oxidative damage, ultimately promoting chromosomal instability, cell cycle arrest, apoptosis, impaired proliferation and/or senescence [1,2]. Additionally, telomere dysfunction can compromise mitochondrial function, causing impaired energy production as well as increased intracellular ROS production [3,4,5]. It is known that telomere attrition occurs in vascular cells from human atherosclerotic lesions, and this erosion leads to cell senescence, accompanied by the acquisition of a pro-inflammatory phenotype [6,7,8]. A large body of evidence in different ethnic populations has reported the association between short telomeres in the leukocytes and coronary artery disease (CAD), as well as the value of telomere length as a predictor of cardiovascular mortality and future coronary events [4,9,10,11]. The rs1333049 (G/C) polymorphism on chromosome 9p21.3 (Chr9p21) is a major genetic risk factor for age-related disorders, notably CAD [12,13]. However, the mechanistic implication of this association is yet not defined [14]. The variant is located on the genomic region near the cell cycle regulating genes, CDKN2B (encoding-p15ink4b) and CDKN2A (encoding-p16ink4a/p14ARF), which are functionally involved in the activation of telomere-directed senescence in human cells [15]. A previous study indicated that the Chr9p21 rs1333049 variant has an impact on p16(INK4a) and p15(INK4b) expression in primary cultures of VSMCs and influences cell proliferation, which likely represents an important mechanism for the association between this genetic locus and susceptibility to CAD [16]. Therefore, it is reasonable to suppose that the Chr9p21 variant may also affect leukocyte telomere length (LTL), providing new knowledge on the role of telomere shortening in atherosclerosis and the potential utility of LTL as a reliable biomarker in a clinical setting. However, despite biological plausibility, there are no studies that have investigated the potential association between Chr9p21variant rs1333049 and telomere shortening in patients with CAD. To address this gap in knowledge, our study aimed to examine the link between the Chr9p21 rs1333049 variant and telomere length shortening as well as their interactive effect on the risk of major adverse cardiovascular events (MACEs) in patients with established CAD.

## 2. Materials and Methods

### 2.1. Study Population

A cohort of 472 patients with angiographically demonstrated and clinically stable CAD (417 males; age 65.2 ± 8.1 years) with both LTL measurements and Chr9p219p21.3 SNP analysis were recruited within the framework of the Italian cohort GENOCOR (Genetic Mapping for Assessment of Cardiovascular Risk). Inclusion criteria included patients affected by a history of CAD with a non-fatal evolution (angina or acute myocardial infarction as first manifestation) with angiographically proven CAD, defined as significant coronary stenosis in at least one diseased vessel (>50% lumen reduction). The severity of CAD was determined by the number of affected vessels (one-, two-, or three-vessel disease). Patients with known malignancy or end-stage renal disease were excluded from this study. The research protocol was approved by the Institution’s Ethics Committee, and all patients gave written informed consent. Classic cardiovascular risk factors were recorded in all patients [17]. Briefly, data were collected on age, gender, diabetes (fasting plasma glucose: >120 mg/dL), hypercholesterolemia (plasma cholesterol: >220 mg/dL), obesity (body mass index: >30 kg/m^2^), and arterial hypertension (systolic blood pressure: >140 mmHg and/or diastolic pressure: >90 mmHg). Smokers were classified as individuals who smoked at least 3 cigarettes per day at the time of analysis, past smokers had quit smoking for at least 6 months, and non-smokers were individuals who had never smoked. Smoking patients were the combined groups of past and current smokers. The Framingham risk score (FRS) was calculated for each patient with an automatic calculator, and the 10-year risk of developing CVD was estimated using baseline measures. We classified the absolute risk percentage as low risk (<10%), medium risk (10–20%), and high risk (>20%) [18]. Data on left ventricular function (LVEF) were obtained using echocardiography or left ventricular angiography.

### 2.2. Follow-Up

All patients were subjected to a follow-up program to certify MACEs, defined as coronary-related death, nonfatal MI, and coronary revascularization (i.e., CABG and PCI). The cause of death was derived from medical records or death certificates provided by local health authorities [17]. The definition of cardiac death required the documentation of either significant arrhythmias, cardiac arrest, or death attributable to congestive heart failure or MI in the absence of any other precipitating factor. All patients were censored after the first adverse cardiovascular event during the follow-up.

### 2.3. Biochemical and Leukocyte Telomere Length Measurements

Blood samples were drawn at recruitment from all patients under fasting conditions and were processed instantly according to standard operating procedures for the assessment of various biochemical parameters: lipid profile (total Cholesterol, LDL cholesterol, HDL cholesterol, and triglycerides), glycemic profile and glycated hemoglobin, cardiac biomarkers (creatine kinase-MB and lactate dehydrogenase), and renal profile (creatinine). In addition, serum uric acid, fibrinnogen, the neutrophil-lymphocyte ratio (NLR), and the platelet-lymphocyte ratio (PLR) were used to evaluate the degree of systemic inflammation. Genomic DNA was extracted from peripheral blood leucocytes by using the QIAGEN BioRobot^®^ EZ1 System. DNA concentration and quality were assessed with a NanoDrop Lite Spectrophotometer (Thermo Scientific, Waltham, MA, USA). An absorbance ratio at both 260 and 280 nm (A260/A280) greater than 1.6 was considered suitable for the subsequent analyses. Genotyping of the Chr9p21 rs1333049 variant was performed using high-resolution melting curve analysis of oligonucleotide fluorescent probes, employing LC Green and a Light Scanner (Idaho Technology, Salt Lake City, UT, USA). LTL was measured by using quantitative real-time methods (CFX384 Touch Real-time PCR detection system, Bio-Rad, Hercules, CA, USA) following previously described protocols [19,20]. Briefly, LTL was measured in genomic DNA by determining the T/S ratio). A relative telomere length was calculated using the equation T/S ratio = 2^−ΔΔCt^, where Ct is a threshold cycle and ΔCt = Ct × telomere − Ct × single copy gene. The T/S ratio reflected the average length of the telomeres across all leukocytes.

### 2.4. Statistical Analyses

Categorical data, expressed as frequencies and percentages, were compared using the Fisher’s exact test. Continuous variables were compared using the Student’s *t*-test and Mann–Whitney U test for parametric and non-parametric data, respectively. The Kruskall–Wallis test was used to compare continuous variables between more than two groups. LTLs were analyzed as both a continuous and categorical variable, stratifying the patients with short and long LTLs (≤median value and >median value, respectively). Categorical variables were compared using the chi-squared test. The Spearman’s rank correlation was used to test the association between LTL and biochemical parameters. Kaplan–Meier survival analysis was performed to compare the difference in survival rate between patients with short and long LTLs and different genotypes using the log rank test. Cox proportional hazard models were used to assess the predictive value of each variable. The interaction between the 9p21.3 variant rs1333049 and LTL on MACE was tested by introducing a corresponding interaction term in the Cox model. Risk estimates were expressed as hazard ratios (HR) with the corresponding 95% confidence intervals (CI). Multivariate Cox proportional hazard analysis was estimated with an adjustment for a range of potential confounders to prevent model over-fitting. Multivariate Cox proportional hazard analysis was estimated with an adjustment for a range of potential confounders. To avoid an over-fitting problem caused by the limited number of events, we restricted the number of covariates that were statistically significant for univariate analysis and/or those that were clinically relevant, including age, gender, smoking, hypertension, hypercholesterolemia, diabetes, previous acute myocardial infarction, and LVEF. A *p*-value of <0.05 was considered statistically significant in this study.

## 3. Results

### 3.1. Baseline Characteristics and LTL

The baseline characteristics of the study population are shown in Table 1.

**Figure 1:**
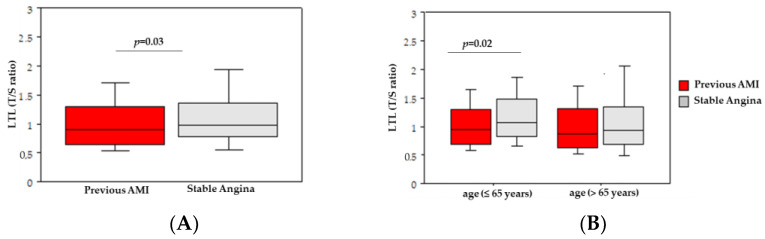
Box-and-whisker plots of leukocyte telomere length (LTL) in patients with previous acute myocardial infarction (AMI) and stable angina (**A**) and in patients stratified by the median of age (**B**). Box-and-whisker plots display interquartile range, median values, and maximum and minimum values.

As expected, a significant correlation was shown between LTL and chronological age (Spearman rho = −0.1, *p* = 0.04). Dichotomized at the median value of LTL, older patients had similarly short LTL compared to younger patients (*p* = 0.02). Gender, smoking status, and other traditional risk factors were not associated with short LTL, with the only exception being hypertension (*p* = 0.02). Also, no significant correlation between LTL and FRS groups was found. LTL was not related to the CAD severity (*p* = 0.79), but patients with previous acute myocardial infarction had a significantly shorter LTL when compared to patients with stable angina (*p* = 0.03), especially for younger subjects (Figure 1).

### 3.2. Relationship between Chr9p21 rs1333049 Variant and LTL

**Figure 2:**
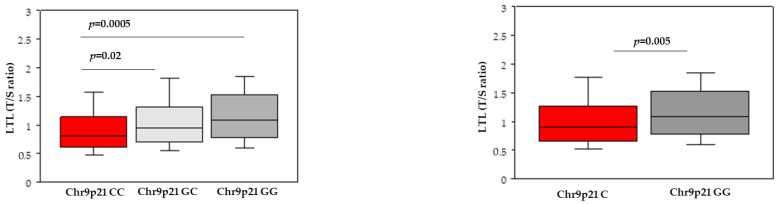
Box-and-whisker plots of leukocyte telomere length (LTL) according to chromosome 9p21.3 (Chr9p21) rs1333049 (G/C) variant.

As regards the genetic analysis, Chr9p21 rs1333049 variant was significantly associated with lower LTL (*p* = 0.0006). Carriers of the CC genotype of this SNP had shorter telomeres than GC carriers (*p* = 0.02) and GG carriers (*p* = 0.0005); no difference was found between GC and GG carriers (Figure 2).

**Figure 3:**
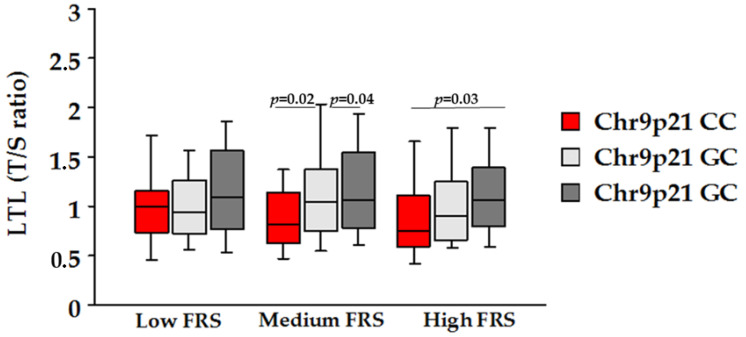
Leukocyte telomere length (LTL) analyzed by genotypes in the low-, intermediate-, and high-risk FRS groups.

The comparison of allele C carriers with GG carriers (AG and AA genotypes combined) also revealed a significant difference (*p* = 0.005). The analysis of biochemical parameters across genotypes revealed no significant differences, with the exception of cholesterol (*p* = 0.03) and LDL (*p* = 0.04) levels in patients carrying the C allele compared to the GG genotype. There was no significant interaction between Chr9p21 rs1333049 variant and common risk factors on LTL. However, the lower value of LTL was observed in carriers of the CC genotype and with high-risk FRS (Figure 3).

On multivariable linear regression, age (*p* = 0.02) and Chr9p21 rs1333049 variant (*p* = 0.002) were the only independent predictors that significantly affected the telomere length among CAD patients (Table 2).

### 3.3. LTL, Chr9p21 rs1333049 Variant, and Clinical Outcome

**Figure 4:**
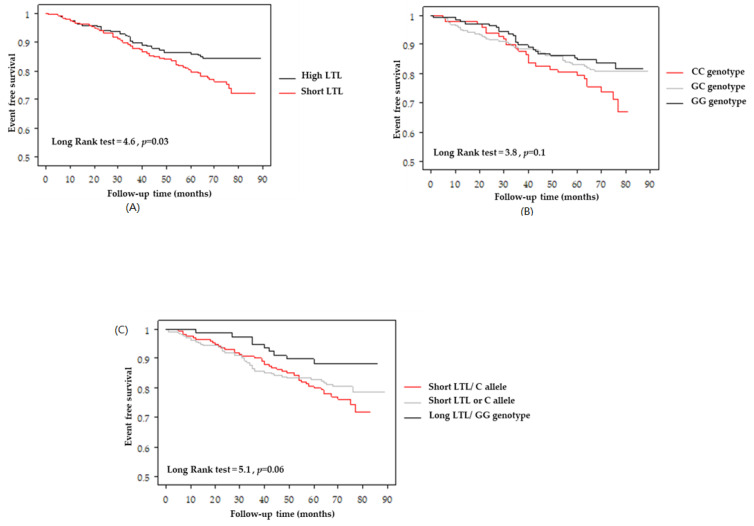
Kaplan–Maier plots of MACEs for LTL stratified as short (≤median value) versus long LTL (>median value) (**A**), of Chr9p21 rs1333049 genotypes (**B**), and stratified in three groups according to LTL and 9p21.3 rs1333049 variant interactions (**C**).

After a follow-up with a mean period of 62 ± 19 months, 90 patients (19.1%) had MACE. Kaplan–Meier survival analyses used to calculate the cumulative probability of MACE with LTL, the carrier status of Chr9p21 rs1333049 variant, and interaction effects are presented in Figure 4.

Short LTL was an independent prognostic factor of MACE incidence (HR: 2.2; 95% CI: 1.3–3.7; *p* = 0.005) after adjustment for age, gender, smoking, hypertension, hypercholesterolemia, diabetes, LVEF, and previous acute myocardial infarction. Neither the Chr9p21 CC genotype (HR: 1.9; 95% CI: 0.9–3.8; *p* = 0.08) or C allele (HR: 1.5; 95% CI: 0.8–2.8; *p* = 0.2) significantly predicted MACE. However, there was significant interaction (*p* = 0.01) between the LTL and Chr9p21 rs1333049 variant (Table 3), with patients with risk-allele C and short LTL having a higher risk (HR:5.8; 95% CI: 1.8–19.2; *p* = 0.004).

## 4. Discussion

This is the first study to report an association between LTL and Chr9p21 rs1333049 variant in a well-characterized cohort of patients with stable CAD. Our findings showed that the baseline telomere length and Chr9p21 rs1333049 variant interactively affect the risk of poor prognosis of patients in our population. Telomeres are considered to be a cellular marker for many aging-related diseases, including cardiovascular disease [21]. In fact, many studies have shown an association between LTL and coronary events [9,10,11], including our previous work, showing that telomere shortening in peripheral cells resulted in an independent predictor of MACEs and all-cause mortality [20]. Additionally, patients carrying both short LTLs and high mtDNA4977 deletion levels had the highest risk of adverse outcome [20], supporting the notion of a mutual crosstalk between mitochondrial damage and telomere dysfunction function in vascular aging and atherosclerosis [5,20]. Although the mechanism of how dysfunctional telomeres promote atherosclerosis is not yet completely clear, critical short LTL induces endothelial and vascular smooth muscle cell senescence, activating a senescence-associated secretory phenotype (SASP), which, in turn, induces pathophysiological cellular and molecular changes such as inflammation, apoptosis, and tissue remodeling [4,5,6,7,8]. To date, the Chr9p21 is the most well-known and replicated genetic marker factor for CAD in different populations, with an estimated 15% to 35% increased risk in carriers of the variant allele in prospective population and case–control [12,13]. However, the GENIUS-CHD consortium did not find an association between genetic variation at Chr9p21 and risk of subsequent CHD event, suggesting the view that chromosome Chr9p21 promotes the atherosclerotic burden rather than subsequent cardiac events [22]. Nevertheless, the mechanism whereby the Chr9p21 locus confers increased susceptibility to coronary atherosclerosis is not yet completely understood.

Of note, the locus seems not to be linked to any conventional CAD risk factors, even if several variants have been reported to influence the lipid profile [23]. However, it may exert its effect through a completely novel biological mechanism related to vascular integrity and atherosclerosis development [24]. Indeed, several studies showed that the Chr9p21 risk variants influence the expression of the non-coding RNA ANRIL and INK4/ARF-associated transcripts (CDKN2A, CDKN2B) [25,26,27,28,29], supporting a crucial role for cell cycle inhibition and key physiological processes, such as vascular senescence and apoptosis in atherosclerosis [24,25,26,27,28]. Despite the role of telomeres in cellular senescence and vascular aging, the relationship between LTL and Chr9p21 on CAD has never been evaluated. Within the current study, we found that LTL was strongly related to Chr9p21 locus variant rs1333049, and their interaction correlated with an increased risk of clinical adverse events in our population of patients with stable CAD. Because SNPs in the Chr9p21 locus have been predicted to influence the expression of CDKN2A/B/ANRIL transcripts [25,26,27,28,29], we can hypothesize that the impairment of p16/Rb and/or p53/p21 pathways may affect the function of cells bearing dysfunctional telomeres (critical telomere shortening), leading to increased senescence and genome instability and, ultimately, promoting the development and progression of atherosclerosis. This concept is also supported by evidence showing that telomere shortening can lead to specific clonal somatic mutations in peripheral leukocytes, which is termed clonal hematopoiesis of indeterminate potential (CHIP) [30,31]. Currently, growing evidence shows that CHIP is a new risk factor for coronary artery disease [32], and it is associated with adverse cardiovascular outcomes [33], representing an exciting new frontier for CAD precision medicine [33]. Collectively, our findings support the ample evidence that telomere attrition (or telomere shortening) is a critical factor in the genesis of vascular aging and atherosclerosis as well as a potential treatment target for CVD in order to inhibit senescent or mutated clonal cells in CAD [34]. There are several limitations to be acknowledged in our study. First, the population consisted exclusively of medically treated patients with stable CAD and had a relatively small sample size. Drug treatment and the low number of patients in studied subgroups may have influenced results with regard to MACE. Second, any influence of other known genetic variants on the Chr9p21 locus was not explored. Third, it is well known that telomere length is regulated and influenced by environmental and genetic factors [2,35], and we cannot exclude the impact of uncontrolled confounders on the interpretation of any associations identified. A combination of further preclinical and clinical studies is required to understand the molecular relationships involving heritable germline variants with genomic instability and acquired somatic mutations, as well as their potential synergistic interactions with known and novel cardiovascular risk factors, including various environmental factors such ionizing radiation and air pollution [35].

## 5. Conclusions

In patients with stable CAD, a strong relationship between LTL and Chr9p21 rs1333049 variant was identified, and they interactively affected the risk of MACE in our population. Treatment approaches, aiming to improve telomere length and dysfunction, could be a novel strategy for the prevention of vascular aging and atherosclerosis development and events.

## Figures and Tables

**Table 1:** Demographic and clinical characteristics of the study population.

Characteristics	Value
Mean age (years ± SD)	65.2 ± 8.1
Males, n (%)	417 (88)
Current smoker, n (%)	303 (64)
Hypertension, n (%)	213 (45)
Diabetes, n (%)	66 (14)
Obesity, n (%)	143 (30)
Hypercholesterolemia, n (%)	327 (69)
LVEF %	51.3 ± 9.5
N° of affected coronaries	
1	231 (49)
2	156 (33)
≥3	85 (18)
Previous myocardial infarction, (%)	274 (58)
Aspirin, n (%)	437 (93)
Statin, n (%)	343 (73)
ACEI/ARB, n (%)	124 (26)
Beta-blocker, n (%)	194 (41)
Calcium channel blocker, n (%)	195 (41)

**Table 2:** Independent effect of the predictor variables for telomere length (natural log-transformed T/S ratio) in a multiple regression analysis.

Variables	β Coefficient	*p* Value
Age	−0.008	0.02
Gender	−0.074	0.4
Glucose	−0.001	0.83
BMI	−0.010	0.18
Total cholesterol	0.150	0.09
LDL cholesterol	−0.150	0.09
HDL cholesterol	−0.154	0.09
Triglycerides	−0.031	0.09
Previous AMI	−0.097	0.07
Creatinine	0.173	0.08
Uric acid	−0.008	0.71
Glycated hemoglobin	0.001	0.9
HOMA	0.001	0.9
NLR	0.001	0.9
PLR	0.001	0.51
LDH	0.001	0.44
CK	0.001	0.77
Fibrinogen	0.001	0.21
9p21.3 rs1333049 variant	−0.107	0.002

**Table 3:** Multivariate Cox proportional hazard model for the risk of major adverse cardiovascular events (MACEs) based on LTL and 9p21.3 rs1333049 variant interactions.

Groups	MACEs HR (95% CI)	*p* Value
**Analysis with 4 groups**		
Long LTL/GG genotypes	1.0	
Long LTL/C allele	3.3 (0.95–11.3)	0.06
Short LTL/GG genotypes	5.3 (1.5–19.3)	0.01
Short LTL/C allele	5.8 (1.8–19.2)	0.004
**Analysis with 3 groups**		
Long LTL/GG genotypes	1.0	
Short LTL or C allele	3.9 (1.2–12.9)	0.03
Short LTL/C allele	5.8 (1.8–19.1)	0.004

## Data Availability

Available upon reasonable request.
